# A Problem in AC Quantized Hall Resistance Measurements and a Proposed Solution

**DOI:** 10.6028/jres.103.038

**Published:** 1998-12-01

**Authors:** M. E. Cage, A. Jeffery

**Affiliations:** National Institute of Standards and Technology, Gaithersburg, MD 20899-0001

**Keywords:** ac quantum Hall effect, capacitance-to-shield, equivalent electrical circuit, longitudinal resistance, multi-series connections, quantized Hall resistance

## Abstract

In all experiments reported to date the measured values of the ac quantized Hall resistances *R*_H_ varied with the frequency of the applied current, and differed significantly from the dc values of *R*_H_, making it difficult to use the ac quantum Hall effect as an absolute impedance standard. We analyze the effects due to the large capacitances-to-shields existing in the sample probes on measurements of *R*_H_ to see if this is the source of the problem. Equivalent electrical circuits are utilized; they contain capacitances and leakage resistances to the sample probe shields, longitudinal resistances within the quantized Hall effect devices, and multiple connections to the devices. The algebraic solutions for the *R*_H_ values in these circuits reveal large out-of-phase contributions to the quantized Hall voltages *V*_H_ that would make it difficult to do accurate measurements with high precision ac bridges. These large out-of-phase contributions could introduce the linear frequency dependences observed in previous *R*_H_ measurements. We predict, however, that quadruple-series connections to the quantum Hall devices yield only small out-of-phase contributions to *V*_H_ which may allow accurate measurements of the quantity *R*_H_ − *R_x_*, where *R_x_* is the longitudinal resistance along the device.

## 1. Background

The quantum Hall effect (QHE) has been successfully used as an intrinsic dc resistance standard. In the integer dc QHE [1–3], the Hall resistance *R*_H_ of the *i*th plateau of a fully-quantized, two-dimensional electron gas (2DEG) is *R*_H_(*i*) = *V*_H_(*i*)/*I*_T_, where *V*_H_(*i*) is the quantum Hall voltage measured between potential probes located on opposite sides of the device, and *I*_T_ is the total current flowing between the source and drain current contacts at the ends of the device. Under ideal conditions, the values of *R*_H_(*i*) in standards-quality devices satisfy the relationships *R*_H_(*i*) = *h*/(*e*^2^*i*) = *R*_K_/*i*, where *h* is the Planck constant, *e* is the elementary charge, *i* is an integer, and *R*_K_ is the von Klitzing constant *R*_K_ ≈ 25 812.807 Ω. However, the conditions are not always ideal. The values of *R*_H_(*i*) can vary with the device temperature *T* and with the frequency *f* of the applied current if it is not dc. Thus the measured values of *R*_H_(*i*) are not necessarily equal to *h*/(*e*^2^*i*).

The current flow within the 2DEG is nearly dissipationless in the quantum Hall plateau regions of high-quality devices, and the longitudinal resistances *R_x_*(*i*) of this standard become very small over ranges of magnetic field that exhibit quantized Hall resistance plateaus. The dc longitudinal resistance is defined to be *R_x_*(*i*) = *V_x_*(*i*)/*I*_T_, where *V_x_*(*i*) is the measured longitudinal voltage drop between potential probes located on the same side of the device.

Many laboratories are now attempting to employ the QHE to realize an intrinsic ac resistance standard by using ac ratio bridges to compare the ac quantized Hall resistances *R*_H_ with ac reference standards. In experiments reported to date [4–9], the measured values of the ac quantized Hall resistances *R*_H_ vary with the applied frequency of the current *f* (usually increasing linearly with *f*), and differ from the dc value of *R*_H_ by at least the factor 10^−7^ *R*_H_(*i*) at a frequency *f* of 1592 Hz (ω = 2π*f* is 10^4^ rad/s). With one exception [10], the reported ac longitudinal resistances *R_x_*(*i*) are significantly larger than the dc longitudinal resistances in the same device under the same temperature and magnetic field conditions. The ac longitudinal resistances increase with increasing frequency of the applied current, and are of order 1 mΩ at 1592 Hz [4,5,11]. The frequency dependences of *R*_H_ and *R_x_* are reported to be a property of the real, resistive (in-phase) component of the ac impedance measurements.

These results might arise from intrinsic properties of the quantum Hall devices. However, in a previous publication [12] we showed that the intrinsic impedance due to the internal Hall capacitance of the two-dimensional electron gas across the QHE device does not account for the observed frequency dependences of the in-phase ac quantized Hall resistances *R*_H_. We also showed [12] that the kinetic inductance of the 2DEG and the magnetic inductance of the device provide no plausible intrinsic impedance explanations for the observed frequency dependences of the in-phase ac longitudinal resistances *R_x_*. Other calculations [13] showed that the intrinsic longitudinal resistances of the QHE device have very little effect on *R*_H_ measurements.

In this paper we investigate what effects the large capacitances-to-shield in the sample probe can have on the measured ac values of *R*_H_.

## 2. Equivalent Electrical Circuit of an AC QHE Standard

We are in the process of deriving exact algebraic solutions for the currents and potentials of equivalent electrical circuits containing (a) multiple connections to the device; (b) capacitances to the sample probe shields; (c) longitudinal resistances within the device; and (d) leakage resistances to the probe shields. Finding the exact algebraic equations for *R*_H_ and *R_x_* is rather difficult, and the solutions are nontrivial.

The exact algebraic solutions for the *R*_H_ values in these circuits reveal large 90° out-of-phase (reactive) contributions to the quantized Hall voltages *V*_H_ that would make it very hard to perform accurate in-phase (resistive) measurements with high precision ac bridges because it is difficult to measure to sufficient accuracy the phase defect (the in-phase or resistive part) of the components used to null the out-of-phase (reactive) signal. Preliminary tests at NIST suggest that these large out-of-phase contributions could introduce linear frequency dependences into the in-phase measurements of *R*_H_.

We find, however, that quadruple-series connections to the quantum Hall devices yield only small out-of-phase contributions to *V*_H_ that should allow accurate measurements of *R*_H_ with small uncertainties. Analyzing these equivalent circuits is a long process, so we present this preliminary report of the results because it may be of use to other laboratories that are preparing, or making, ac QHE measurements.

### 2.1 Circuit Description

**Fig. 1:**
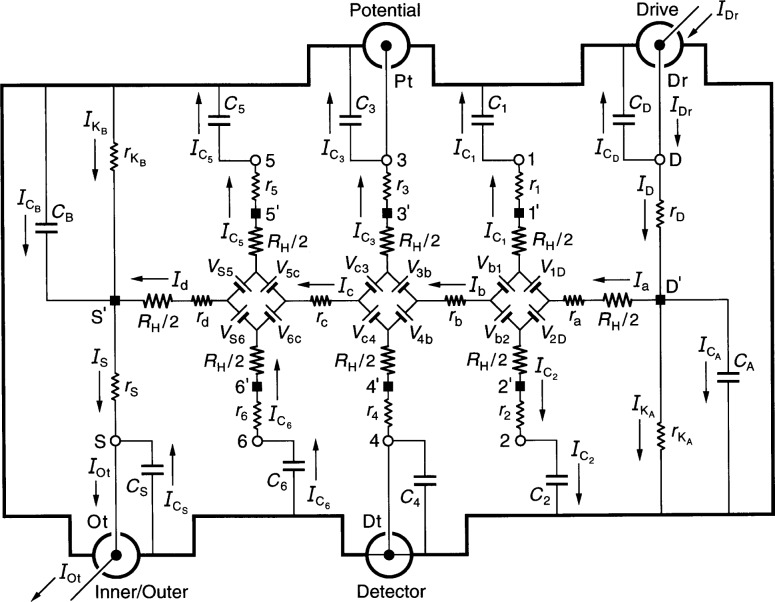
An equivalent electrical circuit representation of a QHE resistance standard while the quantized Hall resistance is being measured in an ac ratio bridge using four-terminal-pair [14,15] measurement techniques. The ac ratio bridge is not shown, nor is the ac reference resistance standard with which the QHE standard is being compared. The symbols are explained in Sec. 2.1. See Sec. 3.1 for the circuit analysis.

Figure 1 shows an equivalent electrical circuit representation of a QHE resistance standard while the standard is being measured in an ac ratio bridge. The quantized Hall resistance *R*_H_(*i*) of the QHE standard is compared with the resistance of an ac reference standard using four-terminal-pair [14,15] measurement techniques. The ac ratio bridge is not shown in the figure, nor is the ac reference standard with which the QHE standard is being compared. Only the QHE standard is shown. This figure is rather detailed, so we explain it one step at a time.

The QHE standard is bounded by an electrical shield indicated by thick lines. This shield is also referred to in the text as outer conductors. To simplify the figure, we label only currents in the inner conductors. A QHE device occupies the central region of the figure. This device is modeled as an equivalent electrical circuit. There are additional components of the ac QHE standard. They are also modeled as circuit elements. These components are described below. The standard has electrical access via coaxial measurement ports labeled Inner/Outer, Detector, Potential, and Drive. (A coaxial port is often referred to in the literature as a terminal-pair.)

The ideal four-terminal-pair measurement definition [14,15] of *R*_H_ is satisfied by the following three simultaneous conditions: (1) The current *I*_Dr_ at the Drive coaxial port is adjusted so that there are no currents in the inner or outer conductors of the Potential coaxial port, i.e., *I*_Pt_ = 0. (2) The potential difference is zero across the inner and outer conductors of the Detector coaxial port. (3) There are no currents in the inner or outer conductors of the Detector coaxial port, i.e., *I*_Dt_ = 0.

It is implicit in the four-terminal-pair definition that the ports are treated as terminal-pairs, and that the current in the inner conductor of every port is equal and opposite to the current in the outer conductor (the shield). Coaxial chokes [16] (located outside the QHE standard and not shown in the figure) assure that the equal/opposite current conditions are satisfied. The current *I*_Ot_ exits the QHE standard at the Inner/Outer port and enters the ac reference standard (not shown).

A short has been drawn in Fig. 1 between the shield and inner conductor at the Detector coaxial port to indicate four-terminal-pair condition (2). We let the Detector potential be zero, i.e., *V*_Dt_ = 0. At bridge balance the quantized Hall resistance *R*_H_(*i*) is defined as

$$V_{\mathrm{Pt}}=[1+\Delta ]R_{H}(i)I_{\mathrm{Ot}},$$ (1)

where *Δ* is the correction factor to *R*_H_(*i*) to be determined in this analysis.

Next we describe the equivalent circuit model of the QHE device. The device has contact pads that provide electrical access to the 2DEG at the source S′, the drain D′, and the potential pads 1′ through 6′. Each contact pad is located at the end of an arm of the device. Every arm in the equivalent circuit has an intrinsic resistor whose value is *R*_H_(*i*)/2. We assume that the device is homogeneous, i.e., that the quantized Hall resistances *R*_H_(*i*) are all measured on plateau regions, that their values are the same on all the Hall potential probe sets, and that they are all measured at the same magnetic flux density value. *R*_H_(*i*) can, however, vary with temperature and frequency.

A positively-charged applied current *I*_a_ enters the 2DEG via device drain contact pad D′, and current *I*_d_ exits the 2DEG via source contact pad S′. The magnetic flux density *B* is directed into the figure. Under these current and magnetic field conditions the drain contact pad D′ and the potential probe contact pads 1′, 3′, and 5′ at the device periphery are at higher potentials than contact pads S′, 2′, 4′, and 6′. These current and flux density directions are chosen to be consistent with those we have used in earlier calculations [12,13,17].

Potentials at the contact pads S′, 1′ through 6′, and D′ are produced by voltage generators *V*_AB_ located between arms A and B of the equivalent circuit. The voltages are defined as

$$V_{\mathrm{AB}}\equiv \frac{R_{H}(i)}{2}|I_{A}\pm I_{B}|,$$ (2)

where *I*_A_ and *I*_B_ are the magnitudes of the current flowing in arms A and B. The currents *I*_A_ and *I*_B_ within the absolute quantity sign of Eq. (2) are added if they both enter or both leave the voltage generator, and are subtracted if one current enters and the other current leaves the generator. For example $V_{1D}=[R_{H}(i)/2]|I_{a}-I_{C_{1}}|$. The voltages generated are functions of *R*_H_(*i*); therefore their values can vary with temperature and frequency.

Diamond-shaped voltage generator arrays of Ricketts and Kemeny [18] are employed in the equivalent circuit, rather than the ring-shaped arrays used first by Delahaye [19] and then by Jeffery, Elmquist, and Cage [20]. The calculations are much simpler with the diamond arrays when longitudinal resistances are included in the circuits [13]. For clarity, the voltage generators are indicated in the figure as batteries, with positive terminals oriented to give the correct potentials along each arm. The ac currents alternate direction, so the voltage generators reverse sign each half cycle. Thus, for the part of the period in which the currents flow in the directions indicated in Fig. 1, the voltage generators have the polarities shown. Half a period later the currents change direction, and all the voltage generators reverse polarities.

The QHE device is mounted at the bottom of a sample probe. Coaxial leads extend from the device contact pads S′, 1′ through 6′, and D′ to connection points S, 1 through 6, and D located outside the cryostat. Each arm of the equivalent circuit has a resistance *r*_S_, *r*_1_ through *r*_6_, or *r*_D_. This resistance includes the contact resistance to the 2DEG, the wire resistance connecting a contact pad on the device to a coaxial sample probe lead, and the inner conductor resistance of that coaxial sample probe lead. The inner conductor lead resistances vary with the liquid helium level in the sample probe. They can be measured pair-wise (using access points S, 1 through 6, and D) as a function of liquid helium level via two-terminal resistance measurements by temporarily replacing the quantum Hall device with electrical shorts at positions S, 1′ through 6′, and D′. The inner conductor coaxial lead resistances are typically each about 1 Ω in ac quantized Hall resistance experiments.

The symbols *r*_a_, *r*_b_, *r*_c_, and *r*_d_ in Fig. 1 represent real (in-phase) longitudinal resistances. Sample probes used in dc QHE measurements have a pair of leads to the source contact pad S′ and another pair to the drain contact pad D′. Only one lead of each pair carries the current, so all four dc resistances *r*_a_, *r*_b_, *r*_c_, and *r*_d_ can be obtained using four-terminal measurements. In order to reduce heat loss, sample probes for the ac QHE have a single coaxial lead to each of the contact pads. Therefore only *r*_b_ and *r*_c_ can be determined directly via ac measurements. Values for *r*_a_ and *r*_d_ could be estimated from their dc *r*_a_/*r*_b_ and *r*_d_/*r*_c_ ratios if the *r*_b_/*r*_c_ ratio is the same for both ac and dc measurements. Typical ac *r*_b_ and *r*_c_ values are reported to be about 1 mΩ at 1592 Hz.

The coaxial leads each have an inner and an outer conductor. The outer conductor coaxial lead resistances are also typically each about 1 Ω in ac quantized Hall resistance experiments. The outer conductors of the coaxial leads are connected together outside the cryostat. They act as electrical shields, and are represented schematically as thick lines in Fig. 1. Large capacitances-to-shield, labeled as *C*_S_, *C*_1_ through *C*_6_, and *C*_D_, exist between the inner and outer conductors of these coaxial leads. The open-circuit capacitances can be measured at points S, 1 through 6, and at D as a function of liquid helium level by temporarily disconnecting the coaxial leads at the device contact pads S′, 1′ through 6′, and D′. The capacitance-to-shield of each coaxial lead in typical ac QHE sample probes is at least 100 pF (1 × 10^−10^ F). A predominately 90° out-of-phase current $I_{C_{S}}$, $I_{C_{1}}$ through $I_{C_{6}}$, or $I_{C_{D}}$ flows through each coaxial lead. These currents have the correct signs in Fig. 1 for this half-cycle.

The outer conductors of the coaxial leads are not the only components of the thick-lined shield in Fig. 1. The grounded outer shell of the sample probe, and variously shaped surfaces placed near the QHE devices have contributed additional inner conductor capacitances-to-shield, *C*_A_ + *C*_B_, in experiments reported to date. The additional capacitances-to-shield are labeled *C*_A_ and *C*_B_, and are placed at either end of the QHE device in the figure. (Note that rather than explicitly using *C*_A_ and *C*_B_, one-eighth of the additional capacitances *C*_A_ + *C*_B_ could instead be added to each of the eight coaxial lead capacitances *C*_S_, *C*_1_ through *C*_6_, and *C*_D_, but that would make the coaxial lead capacitance notation very confusing.) The additional capacitances *C*_A_ + *C*_B_ can be determined by connecting all eight coaxial leads to the device and then measuring the total capacitance-to-shield *C*_T_ at one of the points S, 1 through 6, or D. The total capacitance-to-shield is then *C*_T_ = *C*_S_ + *C*_1_ + *C*_2_ + *C*_3_ + *C*_4_ + *C*_5_ *+ C*_6_ + *C*_D_ + *C*_A_ + *C*_B_, where *C*_A_ = *C*_B_ if the device holder and the wire bonds are symmetrically arranged.

The equivalent circuit accounts for leakage currents between the QHE standard’s inner conductors and the shields via resistances $r_{K_{A}}$ and $r_{K_{B}}$ located on either side of the QHE device. The sample probes should be constructed so these leakage resistances are very large. It would be safest to temporarily replace the device with shorts when measuring the total open-circuit leakage resistance *r*_Lk_ at point S, 1 through 6, or D. If all the contacts are clean, and if the leakage resistances are symmetrically distributed, then $r_{K_{A}}\approx r_{K_{B}}\approx 2r_{\mathrm{Lk}}$ because they are connected in parallel within the circuit.

## 3. Circuit Analyses

We use Kirchoff’s rules to sum the currents at branch points and the voltages around loops to obtain exact algebraic equations for equivalent electrical circuits of ac QHE standards. Finding the exact algebraic solutions for all the currents, and for the correction factor *Δ* as defined by Eq. (1), is rather difficult because there are many coupled equations. Our criteria for obtaining the solutions is that both authors independently derive the same equations, and that computer software verifies the results. It will take time to complete this task, so we present here approximate solutions for some of the currents and for the correction factor *Δ*. Only the largest terms are included in the approximate solutions. However, it was necessary to carry smaller terms in the intermediate approximate equations because the larger terms sometimes unexpectedly canceled.

### 3.1 Single-Series Connections

The ac QHE standard shown in Fig. 1 has one current lead connected to the source contact pad S′ and another current lead connected to the drain pad D′. We refer to this wiring configuration as two single-series connections to the device.

The shunt currents $I_{C_{5}}$, $I_{C_{3}}$, $I_{C_{1}}$, $I_{C_{D}}$, $I_{C_{A}}$, and $I_{K_{A}}$ are much larger than shunt currents $I_{C_{2}}$, $I_{C_{4}}$, $I_{C_{6}}$, $I_{C_{S}}$, $I_{C_{B}}$, and $I_{K_{B}}$ because contact pads 5′, 3′, 1′, and D′ are all near the quantum Hall potential, rather than near the shield potential. The room temperature access points 1, 2, 5, and 6 are open-circuited in the figure. There may be significant antenna noise generated in coaxial leads 1 and 5 because they are near the quantum Hall potential, but we ignore this problem.

We will present the complete list of exact and approximate current solutions in the full paper; only three shunt currents of particular interest are given here:

$$I_{C_{5}}\approx \{[\omega^{2}C_{S}C_{5}R_{H}r_{S}+\omega^{2}C_{5}C_{5}R_{H}r_{5}]+j[\omega C_{5}R_{H}]\}I_{\mathrm{Ot}}$$ (3a)

$$I_{C_{3}}\approx \{-[\omega^{2}C_{3}C_{5}R_{H}R_{H}]+j[\omega C_{3}R_{H}]\}I_{\mathrm{Ot}}$$ (3b)

$$I_{C_{1}}\approx \{-[\omega^{2}C_{1}(C_{3}+C_{5})R_{H}R_{H}]+j[\omega C_{1}R_{H}]\}I_{\mathrm{Ot}}.$$ (3c)

AC QHE experimental values (with cardinal numbers) can be assigned to the circuit elements to estimate the shunt currents. For example, both the *i* = 2 (12 906.4 Ω) and *i* = 4 (6 453.2 Ω) plateaus have been measured in ac experiments, so let *R*_H_ = 10 000 Ω.

$$R_{H}=10^{4}\;\Omega$$ (4a)

$$r_{S}=r_{1}=r_{2}=r_{3}=r_{4}=r_{5}=r_{6}=r_{D}=1\;\Omega$$ (4b)

$$r_{a}=r_{b}=r_{c}=r_{d}=10^{-3}\;\Omega$$ (4c)

$$r_{K_{A}}=r_{K_{B}}=10^{12}\;\Omega$$ (4d)

$$C_{S}=C_{1}=C_{2}=C_{3}=C_{4}=C_{5}=C_{6}=C_{D}=C_{A}=C_{B}=10^{-10}\;F$$ (4e)

$$\omega =10^{4}\;\text{rad/s}.$$ (4f)

Note that, with care, the leakage resistances $r_{K_{A}}$ and $r_{K_{B}}$ can be at least 10^14^, but dirty contacts or poor insulation can make them worse than the 10^12^ Ω assumed in Eq. (4d). The capacitances *C*_A_ and *C*_B_ are difficult to estimate. They may have been rather large in some experiments because grounded disks were placed close to the bonding wires on the device holders, so we let them be 10^−10^ F in Eq. (4e).

**Fig. 2:**
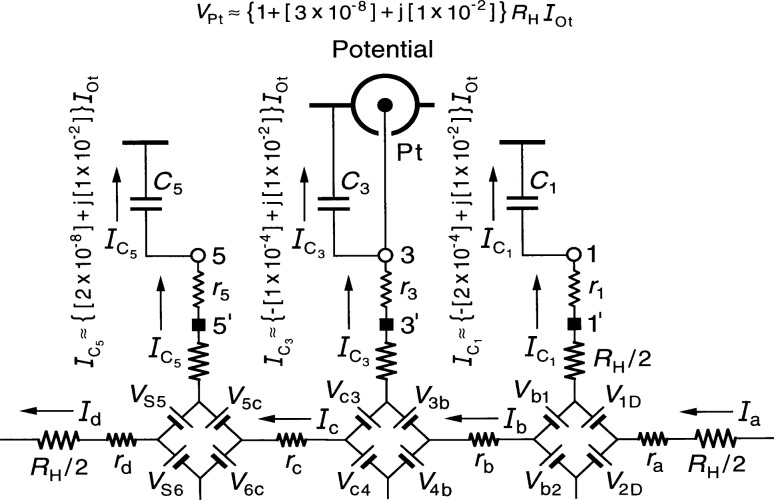
Enlargement of Fig. 1 in the vicinity of the Potential coaxial port, plus the approximate numerical values of $I_{C_{5}}$, $I_{C_{3}}$, $I_{C_{1}}$, and *V*_Pt_ calculated from Eqs. (3a) to (3c) and Eq. (5b).

Figure 2 shows an enlargement of Fig. 1 in the vicinity of the Potential coaxial port, plus the approximate numerical values of $I_{C_{5}}$, $I_{C_{3}}$, and $I_{C_{1}}$ calculated from Eqs. (3a) to (3c). Note that a 1 % out-of-phase current passes through each of the coaxial cable capacitances *C*_5_, *C*_3_, and *C*_1_ in this example (and also through *C*_D_ and *C*_A_). That is not necessarily a problem if the bridge drive can provide this extra 5 % of 908 out-of-phase current to *I*_Dr_. It is a problem, however, if these out-of-phase currents generate unwanted signals in the quantized Hall voltage *V*_H_.

*V*_H_ = *V*_Pt_ is obtained by summing the voltages between the inner conductors of the Detector coaxial port and the Potential coaxial port; taking the path through arm 4, voltage generators *V*_c4_ and *V*_c3_, and arm 3 we find that

$$V_{\mathrm{Pt}}=R_{H}I_{c}-r_{3}I_{C_{3}},$$ (5a)

or approximately

$$\begin{array}{c} V_{\mathrm{Pt}}\approx \{[1+\omega^{2}C_{S}C_{5}R_{H}r_{S}+\omega^{2}C_{3}C_{5}R_{H}r_{3}+\omega^{2}C_{5}C_{5}R_{H}r_{5}]\\ +j[\omega C_{5}R_{H}]\}R_{H}I_{\mathrm{Ot}} \end{array}$$ (5b)

$$V_{\mathrm{Pt}}=[1+\Delta ]R_{H}(i)I_{\mathrm{Ot}}.$$ (5c)

The numerical value of *V*_Pt_ in Eq. (5b) for this typical experimental example is

$$V_{\mathrm{Pt}}\approx \{1+[3\;\times \;10^{-8}]+j[1\;\times \;10^{-2}]\}R_{H}I_{\mathrm{Ot}}.$$ (6)

The 3 × 10^−8^ correction factor in the real part of the *V*_Pt_ signal is quite large compared with the 2.4 × 10^−8^ relative combined standard uncertainty of the complete measurement sequence at NIST [21] between the quantized Hall resistance and the calculable capacitor. This measurement chain is used to assign an SI value to *R*_H_ and to realize the ohm. The ac QHE would replace or verify parts of this measurement chain only if the uncertainty is small enough. However, a correction having a small uncertainty could probably be made to *V*_Pt_ with careful measurements of the circuit elements in Fig. 1 and by using Eq. (5b) to calculate the correction factor.

A much more serious problem is the 1 % contribution to *V*_Pt_ in the out-of-phase signal. High precision ac bridges at NIST are not capable of providing accurate measurements of *V*_Pt_ if the out-of-phase signal is larger than 10^−5^ times the in-phase signal. Even at the 1 × 10^−5^ level, accurate measurements can be done only with great difficulty because care must be taken to correct for the in-phase (phase defect) contributions of the bridge components used to null the out-of-phase *V*_Pt_ signal. The in-phase (phase defect) signals of these components can be unintentionally added to the real, in-phase components of *V*_Pt_. Preliminary tests with a NIST bridge suggest that the in-phase (phase defect) signals due to the out-of-phase components could vary linearly with *ω* (as observed in many ac QHE experiments). These phase defect contributions are in addition to the normal second-order terms in the in-phase part of the signal that vary with *ω*^2^, such as the *ω*^2^*C*_S_*C*_5_*R*_H_*r*_S_, *ω*^2^*C*_3_*C*_5_*R*_H_*r*_3_, and *ω*^2^*C*_5_*C*_5_*R*_H_*r*_5_ terms in Eq. (5b).

Equation (5b) predicts that the out-of-phase term j[*ωC*_5_*R*_H_] in the expression for *Δ* can be reduced by disconnecting coaxial cable 5 at position 5′, where 5′ is either located at the potential contact pad on the QHE device, or at an intermediate contact point on the sample holder. Several problems occur with this approach, however. There is a capacitance *C*_5′_ between the QHE device and the shield. (This capacitance replaces capacitance *C*_5_ in Fig. 1, while *r*_5_ remains unchanged because the shield resistance is typically also about 1 Ω.) *C*_5′_ is certainly much smaller than *C*_5_, so the out-of-phase term is much smaller, but does it reduce the out-of-phase term enough, and how can the capacitance *C*_5_*_'_* be measured since the coaxial lead is disconnected? Disconnecting cable 5 also precludes measuring the quantized Hall resistance with reversed magnetic field direction. All ac QHE experiments have found that these problems make the single-series wiring configuration unacceptable.

### 3.2 Double-Series Connections

**Fig. 3:**
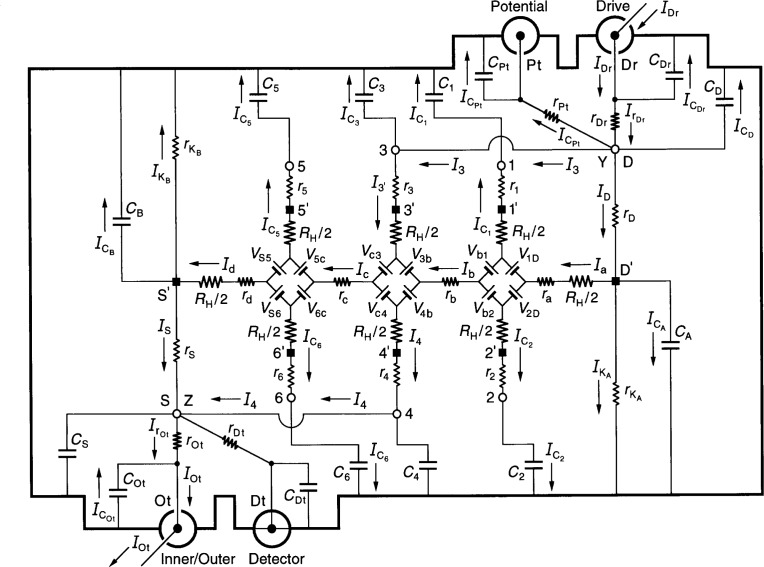
An equivalent electrical circuit representation of a QHE resistance standard with two double-series connections while the standard is being measured in an ac ratio bridge using four-terminal-pair measurement techniques. See Sec. 3.2 for the circuit analysis.

Figure 3 shows an ac QHE standard with two double-series connections to the device. These connections were first used by Delahaye [4] to overcome the above problems. All subsequent experiments have used double-series or triple-series connections.

Short coaxial leads outside the cryostat connect room temperature access points 3 and D at point Y. Two other short coaxial leads connect access points 4 and S at point Z. Short coaxial leads connect point Y with the Drive and Potential ports, and point Z with the Inner/Outer and Detector ports. Typical values for the extra circuit elements are

$$r_{\mathrm{Ot}}=r_{\mathrm{Dt}}=r_{\mathrm{Pt}}=r_{\mathrm{Dr}}=10^{-3}\;\Omega$$ (7a)

$$C_{\mathrm{Ot}}=C_{\mathrm{Dt}}=C_{\mathrm{Pt}}=C_{\mathrm{Dr}}=10^{-12}\;F.$$ (7b)

The other circuit elements of Fig. 3 have the typical values listed in Eqs. (4a) to (4f).

Four currents are of particular interest. Their approximate solutions are

$$\begin{array}{c} I_{C_{5}}\approx \{-[\omega^{2}C_{5}C_{B}R_{H}r_{S}-\omega^{2}C_{5}C_{5}R_{H}r_{5}+\omega^{2}C_{5}C_{6}R_{H}r_{5}]\\ +j[\omega C_{5}R_{H}]\}I_{\mathrm{Ot}} \end{array}$$ (8a)

$$I_{C_{3}}\approx \{-[\omega^{2}C_{3}C_{5}R_{H}R_{H}]+j[\omega C_{3}R_{H}]\}I_{\mathrm{Ot}}$$ (8b)

$$\begin{array}{c} I_{C_{1}}\approx \{-[-\omega^{2}C_{1}(C_{1}-C_{5})R_{H}R_{H}+\omega^{2}C_{1}C_{6}R_{H}r_{S}]\\ +j[\omega C_{1}R_{H}]\}I_{\mathrm{Ot}} \end{array}$$ (8c)

$$I_{3\prime }\approx \left\{\left[\frac{r_{D}}{R_{H}}\right]\right\}I_{\mathrm{Ot}}+I_{C_{1}}.$$ (8d)

**Fig. 4:**
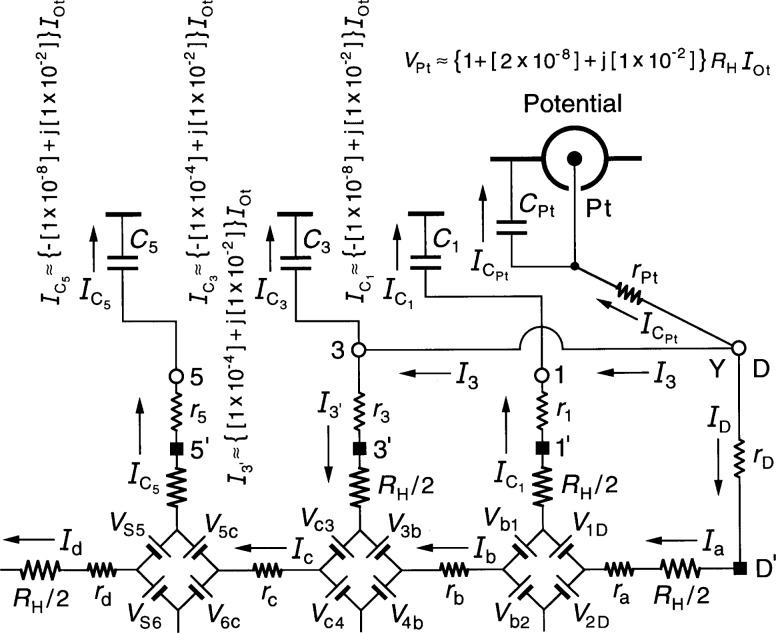
Enlargement of Fig. 3 in the vicinity of the Potential coaxial port, plus the approximate numerical values of $I_{C_{5}}$, $I_{C_{3}}$, *I*_3′_, $I_{C_{1}}$, and *V*_Pt_ calculated from Eqs. (8a) to (8d) and Eq. (9b).

Figure 4 shows an enlargement of Fig. 3 in the vicinity of the Potential coaxial port, plus the approximate numerical values of $I_{C_{5}}$, $I_{C_{3}}$, *I*_3′_, and $I_{C_{1}}$ calculated from Eqs. (8a) to (8d). A 1 % out-of-phase current still passes through each of the coaxial cable capacitances *C*_5_, *C*_3_, and *C*_1_ in this example, and also through resistances *R*_H_/2 and *r*_3_ of arm 3. The current $I_{C_{1}}$ enters the Drive, goes to point Y, to point 3, through arm 3, through *r*_b_, through arm 1, and then exits through *C*_1_. The current $I_{C_{5}}$ enters the Drive, goes to point Y, to point D′, through *r*_a_, *r*_b_, and *r*_c_ through arm 5, and then exits through *C*_5_. The current $I_{C_{3}}$ enters the Drive, goes to point Y, to point 3, and then exits through *C*_3_.

*V*_H_ = *V*_Pt_ is obtained by summing the voltages between the inner conductors of the Detector coaxial port and the Potential coaxial port. Taking the path through arms 3 and 4 of Fig. 3 we find

$$V_{\mathrm{Pt}}=R_{H}I_{c}+R_{H}I_{4}+r_{4}I_{4}+r_{3}I_{3\prime }-r_{\mathrm{Pt}}I_{C_{\mathrm{Pt}}},$$ (9a)

or approximately

$$V_{\mathrm{Pt}}\approx \left\{\left[1+\frac{r_{3}r_{D}}{R_{H}R_{H}}+\frac{r_{4}r_{S}}{R_{H}R_{H}}\right]+j[\omega C_{5}R_{H}]\right\}R_{H}I_{\mathrm{Ot}}$$ (9b)

$$V_{\mathrm{Pt}}=[1+\Delta ]R_{H}(i)I_{\mathrm{Ot}}.$$ (9c)

The numerical value of *V*_Pt_ in Eq. (9b) for this typical experimental example is

$$V_{\mathrm{Pt}}\approx \{1+[2\;\times \;10^{-8}]+j[1\;\times \;10^{-2}]\}R_{H}I_{\mathrm{Ot}}.$$ (10)

The real part of *V*_Pt_ is just the dc double-series prediction [13]. Unlike Eq. (5b) for the single-series solution, it has no significant second-order *ω*^2^ terms. However, there is still the 1 % out-of-phase problem in the j[*ωC*_5_*R*_H_] term of *Δ* that would make it very difficult to perform accurate measurements, due to large phase defects of the components used to null the out-of-phase signal. Also, the same problems discussed at the end of Sec. 3.1 occur if coaxial leads 5 and 1 are disconnected at points 5′ and 1′.

We have not yet analyzed a triple-series circuit with short coaxial leads added between points Y and 1, and between Z and 6. However, it seems clear that the large out-of-phase j[*ωC*_5_*R*_H_] term would still appear in the *V*_Pt_ equation, giving the same measurement problems.

### 3.3 Quadruple-Series Connections

**Fig. 5:**
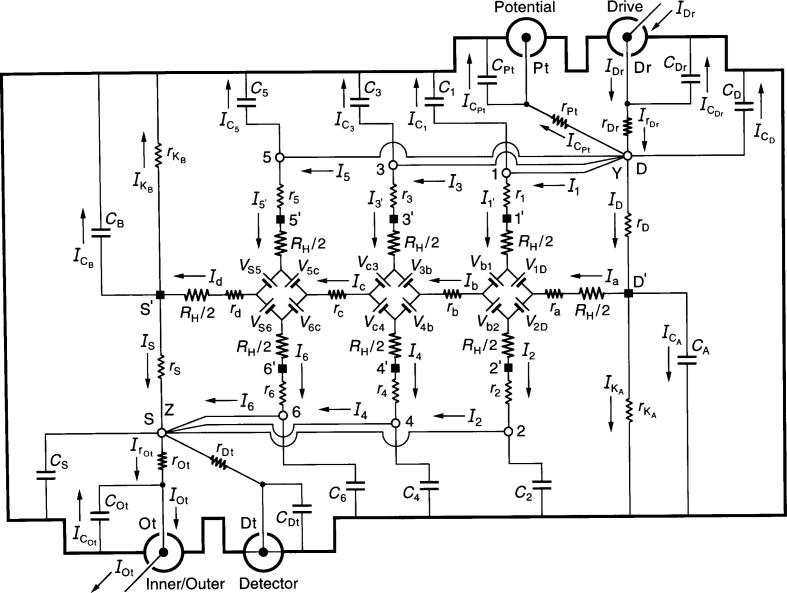
An equivalent electrical circuit representation of a QHE resistance standard with two quadruple-series connections while the standard is being measured in an ac ratio bridge using four-terminal-pair measurement techniques. See Sec. 3.3 for the circuit analysis.

Figure 5 shows an ac QHE standard with two quadruple-series connections to the device. The circuit elements have the same typical values given by Eqs. (4a) to (4f) and Eqs. (7a) to (7b).

Six currents are of particular interest. Their approximate solutions are

$$I_{C_{5}}\approx \{-[\omega^{2}C_{5}C_{B}R_{H}r_{S}]+j[\omega C_{5}R_{H}]\}I_{\mathrm{Ot}}$$ (11a)

$$I_{5\prime }\approx \left\{\left[\frac{r_{c}}{R_{H}}\right]+j\left[\frac{r_{c}}{R_{H}}\omega C_{B}r_{S}\right]\right\}I_{\mathrm{Ot}}$$ (11b)

$$I_{C_{3}}\approx \{-[\omega^{2}C_{3}C_{B}R_{H}r_{S}]+j[\omega C_{3}R_{H}]\}I_{\mathrm{Ot}}$$ (11c)

$$\begin{array}{c} I_{3\prime }\approx \{\left[\frac{r_{b}}{R_{H}}+\frac{r_{D}r_{1}}{R_{H}R_{H}}\right]\\ +j\left[\frac{r_{D}}{R_{H}}\frac{r_{1}}{R_{H}}\omega C_{A}R_{H}+\frac{r_{b}}{R_{H}}\omega C_{B}r_{S}\right]\}I_{\mathrm{Ot}} \end{array}$$ (11d)

$$I_{C_{1}}\approx \{-[\omega^{2}C_{1}C_{B}R_{H}r_{S}]+j[\omega C_{1}R_{H}]\}I_{\mathrm{Ot}}$$ (11e)

$$I_{1\prime }\approx \left\{\left[\frac{r_{D}}{R_{H}}\right]+j\left[\frac{r_{D}}{R_{H}}\omega C_{A}R_{H}\right]\right\}I_{\mathrm{Ot}}.$$ (11f)

**Fig. 6:**
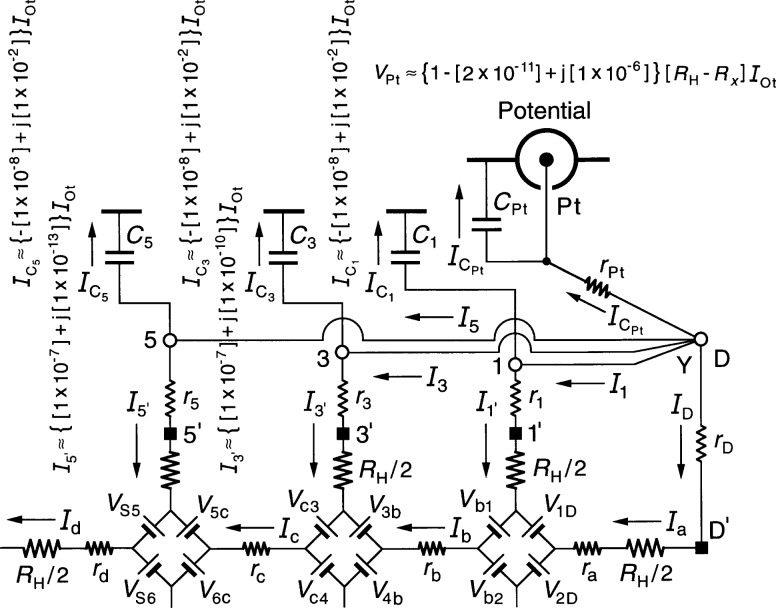
Enlargement of Fig. 5 in the vicinity of the Potential coaxial port, plus the approximate numerical values of $I_{C_{5}}$, $I_{5}\prime$, $I_{C_{3}}$, $I_{3\prime }$, $I_{C_{1}}$, and *V*_Pt_ calculated from Eqs. (11a) to (11e) and Eq. (12b).

Figure 6 shows an enlargement of Fig. 5 in the vicinity of the Potential port, plus the approximate numerical values of $I_{C_{5}}$, *I*_5′_, $I_{C_{3}}$, *I*_3′_, and $I_{C_{1}}$ calculated from Eqs. (11a) to (11e). A 1 % out-of-phase current still passes through each of the coaxial cable capacitances *C*_5_, *C*_3_, and *C*_1_ in this example. However, these currents all enter the Drive, go to point Y, to point 3, and then out through the capacitors, bypassing the arms 5, 3, and 1.

*V*_H_ = *V*_Pt_ is obtained by summing the voltages between the inner conductors of the Detector coaxial port and the Potential coaxial port. Taking the path through arms 3 and 4 of Fig. 5 we find

$$V_{\mathrm{Pt}}=R_{H}I_{c}+(R_{H}+r_{4})I_{4}+r_{3}I_{3\prime }-r_{\mathrm{Pt}}I_{C_{\mathrm{Pt}}},$$ (12a)

or approximately

$$\begin{array}{c} V_{\mathrm{Pt}}\approx \{[1-\frac{\left(r_{b}+r_{c}\right)}{R_{H}}+\left(\frac{r_{3}r_{b}}{R_{H}R_{H}}+\frac{r_{4}r_{c}}{R_{H}R_{H}}\right)\\ +\left(\frac{r_{S}r_{4}r_{6}}{R_{H}R_{H}R_{H}}+\frac{r_{D}r_{1}r_{3}}{R_{H}R_{H}R_{H}}\right)]+j[\omega C_{B}r_{S}]\}R_{H}I_{\mathrm{Ot}}. \end{array}$$ (12b)

The numerical value of *V*_Pt_ in Eq. (12b) for this typical experimental example is

$$V_{\mathrm{Pt}}\approx \{1-[1.999\;78\times 10^{-7}]+j[1\times 10^{-6}]\}R_{H}I_{\mathrm{Ot}}.$$ (13)

The real part of *V*_Pt_ appears to be an order of magnitude larger than the double-series prediction, but again with no significant second-order *ω*^2^ terms. However, *V*_Pt_ actually measures the quantized Hall voltage *V*_H_ across the device *minus* the longitudinal voltage *V_x_*(2,6) along the device between points 2 and 6; i.e.,

$$V_{\mathrm{Pt}}=[1+\Delta ][R_{H}(i)-R_{x}(2,6)]I_{\mathrm{Ot}},$$ (14)

where *R_x_*(2,6) is the longitudinal resistance (*r*_b_ + *r*_c_). The quantity [*R*_H_(*i*) − *R_x_*(2,6)] has a correction factor *Δ* that is only 2.2 × 10^−11^ for this example.

There is only a 1 × 10^−6^ out-of-phase component in the *V*_Pt_ signal for the numerical example given in Eq. (13). The size of this j[*ωC*_B_*r*_S_]*R*_H_*I*_Ot_ component is much smaller than the j[*ωC*_5_*R*_H_]*R*_H_*I*_Ot_ component in Eqs. (5b) and (9b) because the device-side of capacitor *C*_B_ is near zero potential, rather than near the quantum Hall potential of the device-side of capacitor *C*_5_. This small out-of-phase component can be handled, with care, by the NIST ac bridges; it can be further reduced by minimizing the capacitance-to-shield *C*_B_. We suggest that future experiments surround the QHE device with a continuous conducting shield in the shape of a cylindrical pillbox to form reproducible capacitances *C*_A_ and *C*_B_, and then support the device in the center of the largest possible pillbox to minimize these capacitances.

## 4. Summary

We are in the process of analyzing the effects of large capacitances-to-shields in sample probes on measurements of the quantized Hall resistance *R*_H_ using equivalent electrical circuits with capacitances and leakage resistances to the sample probe shields, longitudinal resistances within the quantized Hall effect devices, and multiple connections to the devices. The exact algebraic solutions for the *R*_H_ values in these circuits reveal large 908 out-of-phase contributions to the quantized Hall voltages *V*_H_ that would make it very difficult to make accurate measurements with high precision ac bridges for single-series, double-series, and triple-series connections to the QHE devices.

We predict, however, that quadruple-series connections to the devices yield only small out-of-phase contributions to *V*_H_ that should allow accurate determinations with small uncertainties of the quantity [*R*_H_ − *R_x_*(2,6)], where *R_x_*(2,6) is the longitudinal resistance along the device. It is unfortunate that the quantity [*R*_H_ − *R_x_*(2,6)] is obtained, rather than *R*_H_, but the fact that all eight coaxial leads remain connected to the device contact pads means that the values of all the circuit elements in the equivalent circuit representation of the QHE standard could be determined. It also means that both ac and dc measurements of *V*_H_ and *V_x_* could be performed on the same cool-down, for both magnetic field directions.
